# Supplementary material for: Disability-Related Mortality Inequity in South Korea: Comparison in Terms of Seoul Metropolitan/Non-Metropolitan Areas and Income Levels
Source: Healthcare (Basel). 2024 Jan 23;12(3):293. doi: 10.3390/healthcare12030293 (PMC10855041; doi:10.3390/healthcare12030293)
Supplement: Supplementary file 1 [file healthcare-12-00293-s001.zip › healthcare-2751641-supplementary.pdf]

Table S1. General characteristics of the entire population of people with disabilities

Unit: Total population

		Metropolitan area			Non-metropolitan area			Total
		High income (%)	Median income (%)	Low income (%)	High income (%)	Median income (%)	Low income (%)	
Disability type	Physical	120,267 (60.5)	103,540 (60.2)	95,723 (48.5)	178,718 (59.2)	139,001(59.6)	133,792 (45.9)	771,041
	Brain lesions	11,498 (5.8)	9,690 (5.6)	13,669 (6.9)	17,898 (5.9)	13,480 (5.8)	20,529 (7.0)	86,764
	Visual/hearing/speech	53,608 (26.9)	44,377 (25.8)	48,042 (24.3)	88,481 (29.3)	63,282 (27.1)	65,636 (22.5)	363,426
	Developmental	2,156 (1.1)	3,593 (2.1)	19,392 (9.8)	2,867 (0.9)	5,353 (2.3)	36,443 (12.5)	69,804
	Kidney	5,338 (2.7)	4,869 (2.8)	5,336 (2.7)	6,250 (2.1)	5,121 (2.2)	6,416 (2.2)	33,330
	Others	6078 (3.1)	5,822 (3.4)	15,137 (7.7)	7,735 (2.6)	7,089 (3.0)	28,703 (9.8)	70,564
Age, years	0–9	-	17(0.0)	82 (0.0)	3 (0.0)	27 (0.0)	89(0.0)	218
	10–19	1,319 (0.7)	3,574 (2.1)	7,982 (4.0)	1,338 (0.4)	3,653 (1.6)	9,556 (3.3)	27,422
	20–29	5,585 (2.8)	8,872 (5.2)	8,786 (4.5)	5,537 (1.8)	9,518 (4.1)	12,779 (4.4)	51,077
	30–39	20,291 (10.2)	19,947 (11.6)	21,911 (11.1)	22,822 (7.6)	23,462 (10.1)	35,679 (12.2)	144,112
	40–49	35,279 (17.7)	40,549 (23.6)	44,038 (22.3)	43,565 (14.4)	48,340 (20.7)	67,742 (23.2)	279,513
	50–59	47,157 (23.7)	52,268 (30.4)	58,560 (29.7)	70,203 (23.2)	71,462 (30.6)	82,138 (28.2)	381,788
	60–69	64,122 (32.2)	34,686 (20.2)	42,081 (21.3)	108,097 (35.8)	54,183 (23.2)	55,767 (19.1)	358,936
	>70	25,192(12.7)	11,978 (7.0)	13,859 (7.0)	50,384 (16.7)	22,681 (9.7)	27,769 (9.5)	151,863
Sex	Male	119,573(60.1)	110,302 (64.2)	112,950 (57.2)	174,567 (57.8)	145,490 (62.4)	163,215 (56.0)	826,097
	Female	79,372 (39.9)	61,589 (35.8)	84,349 (42.8)	127,382 (42.2)	87,836 (37.6)	128,304 (44.0)	568,832
BMI, kg/m^2	Normal (18.5–25)	102,234	89,255 (51.9)	101,776 (51.6)	162,516 (53.8)	126,231 (54.1)	154,137(52.9)	736,149
	Overweight (25–29)	77,987 (39.2)	64,928 (37.8)	71,163 (36.1)	113,008 (37.4)	84,799 (36.3)	100,343 (34.4)	512,228
	Obese (≥30)	14,115 (7.1)	13,220 (7.7)	16,760 (8.5)	18,037 (6.0)	15,543 (6.7)	23,246 (8.0)	100,921

	Underweight (<18.5)	4,609 (2.3)	4,488 (2.6)	7,600 (3.9)	8,388 (2.8)	6,753 (2.9)	13,793 (4.7)	45,631
Smoking	Non-smoker	126,808 (63.7)	101,473 (59.0)	135,881 (68.9)	210,309 (69.7)	150,684 (64.6)	217,004 (74.4)	942,159
	Former smoker	49,045 (24.7)	47,097 (27.4)	41,178 (20.9)	59,028 (19.5)	53,293 (22.8)	48,400 (16.6)	298,041
	Smoker	14,935 (7.5)	15,208 (8.8)	12,865 (6.5)	18,742 (6.2)	17,599 (7.5)	15,466 (5.3)	94,815
Drinking (number of days per week)	0	8,157 (4.1)	8,113 (4.7)	7,375 (3.7)	13,870 (4.6)	11,750 (5.0)	10,649 (3.7)	59,914
	1–2	120,794 (60.7)	95,146 (55.4)	123,662 (62.7)	199,269 (66.0)	140,798 (60.3)	193,370 (66.3)	873,039
	3–4	49,423 (24.8)	40,174 (23.4)	37,226 (18.9)	64,093 (21.2)	47,635 (20.4)	43,466 (14.9)	282,017
	≥5	28,728 (14.4)	36,571 (21.3)	36,411 (18.5)	38,587 (12.8)	44,893 (19.2)	54,683 (18.8)	239,873
Mortality status	Non-mortality	194,916 (98.0)	168,841(98.2)	193,227 (97.9)	293,547 (97.2)	227,977 (97.7)	283,269 (97.2)	1,361,777
	Mortality	4,029 (2.0)	3,050 (1.8)	4,072 (2.1)	8,402(2.8)	5,349 (2.3)	8,250 (2.8)	33,152
Total		198,945(100)	171,891	197,299 (100.0)	301,949 (100)	233,326 (100.0)	291,519(100.0)	1,394,929
